# Metabolic Syndrome in Rheumatoid Arthritis

**DOI:** 10.1155/2013/710928

**Published:** 2013-01-30

**Authors:** Iván Ferraz-Amaro, Carlos González-Juanatey, Raquel López-Mejias, Leyre Riancho-Zarrabeitia, Miguel A. González-Gay

**Affiliations:** ^1^Division of Rheumatology, Hospital Universitario de Canarias, 38320 Santa Cruz de Tenerife, Spain; ^2^Division of Cardiology, Lucus Augusti University Hospital, 27004 Lugo, Spain; ^3^Division of Rheumatology, Hospital Universitario Marqués de Valdecilla, IFIMAV, Avenida de Valdecilla s/n, 39008 Santander, Spain

## Abstract

Insulin resistance is an essential feature of the metabolic syndrome that has been linked to rheumatoid arthritis (RA). Understanding how inflammation arising in one tissue affects the physiology and pathology of other organs remains an unanswered question with therapeutic implications for chronic conditions including obesity, diabetes mellitus, atherosclerosis, and RA. Adipokines may play a role in the development of atherogenesis in patients with RA. Biologic therapies, such as TNF-**α** antagonists, that block proinflammatory cytokines have beneficial effects on the insulin resistance that is often observed in patients with RA.

## 1. What Is Metabolic Syndrome?

The term “metabolic syndrome” (MS) refers to a clustering of specific cardiovascular (CV) disease risk factors including central obesity, hypertension, high triglycerides, and low HDL levels whose underlying pathophysiology is thought to be related to insulin resistance [1]. Some clinical studies [2] debate whether MS is a distinct pathophysiologic entity or simply reflects an association of CV risk factors, while others [3] argue that each individual component of the MS confers an increased risk of CV-related death but this risk is more pronounced when the MS itself is present. Nevertheless, it is thought that the more components of the MS that are evident, the higher is the CV mortality rate, and many studies have shown that patients diagnosed with the MS have more prevalent CV disease or are at greater risk of developing CV complications. 

It is estimated that around a quarter of the world's adult population have MS [4] and they are twice as likely to die from and three times as likely to have a heart attack or stroke compared with people without the syndrome [3]. People with MS have a fivefold greater risk of developing type 2 diabetes [5]. In addition, MS has also been associated with several obesity-related disorders including fatty liver disease with steatosis, fibrosis and cirrhosis [6], hepatocellular and intrahepatic cholangiocarcinoma, chronic kidney disease [7], polycystic ovary syndrome [8], sleep-disordered breathing, including obstructive sleep apnea [9], and hyperuricemia and gout [10].

There are five definitions for the MS [11–14] (Table 1). However, the relative value of the different metabolic syndrome definitions in terms of prognosis and management has been established to be similar. For example, when data from the Framingham population were examined using ATPIII, IDF, and EGIR definitions of the MS, associations for incident type 2 diabetes and for CV disease were found to be equivalent [15]. Nevertheless, the National Cholesterol Education Program (NCEP/ATPIII) and International Diabetes Federation (IDF) definitions are the most widely used. The WHO, ATPIII, and IDF definitions include type 2 diabetes as syndrome traits. Experts do not all agree that type 2 diabetes should be part of the definition, as the importance of the syndrome is that it identifies patients at increased risk for the development of diabetes. 

In summary, an MS indicates the presence of a series of risk factors that are associated with the risk of CV complications.

## 2. Metabolic Syndrome and Rheumatoid Arthritis

Rheumatoid arthritis (RA) is a chronic, systemic, inflammatory disorder of unknown etiology that if uncontrolled may lead to destruction and deformity of joints due to erosion of cartilage and bone. Epidemiologic data suggest that RA is an independent risk factor for CV disease [26, 27]. The development of accelerated atherosclerosis and increased risk of CV disease in patients with RA may be influenced by the occurrence of MS [28]. An association between inflammatory activity of RA and MS has also been suggested (Table 2). Also high incidence of MS has been reported in patients with RA. With respect to this, in a series of 283 patients and 226 controls, Da Cunha et al. [16] reported that 39% of RA patients met criteria for MS while these criteria were only fulfilled in 19% of controls (*P* = 0.001). These authors also found increased prevalence of waist circumference, elevated blood pressure, and increased fasting glucose in this series of RA patients when compared with controls. In this study the risk of having MS was significantly higher in RA patients than controls (odds ratio (OR) = 1.87 (95% confidence interval (CI) = 1.17–3.00), *P* = 0.01); and disease activity score-28 (DAS28) was significantly higher in RA patients with MS than in those without MS (3.59 ± 1.27  versus  3.14 ± 1.53; *P* = 0.01). Disease duration, the presence of rheumatoid factor, and extra-articular manifestations were similar in RA patients with and without MS in this study. Nevertheless, the frequency of MS in RA varies according to the criteria used for the assessment. In this regard, using the WHO criteria in 154 patients with RA and 85 controls, Chung et al. [17] observed the presence of MS in 42% of RA patients with long-standing disease, in 31% of RA patients with early arthritis, and in 11% of the controls. In the same study, when NCEP criteria were used, the prevalence of MS was 30% in RA patients with long-standing disease, 22% in patients with RA patients with early arthritis and 10% in controls, respectively. In this paper, coronary-artery atherosclerosis was studied by electron beam computed tomography, and RA patients with MS were found to have a higher coronary-artery calcification score (OR = 2.02 (95% CI 1.03–3.97), *P* = 0.04). 

The association of RA and MS was confirmed in patients with short disease duration. With respect to this, Dao et al. [18] assessed the presence of MS in 105 women with RA and disease duration less than 3 years and 105 age-matched healthy women. Different definitions for MS were tested in this study (Joint Consensus, International Diabetes Federation, National Cholesterol Education Program 2004 and 2001, European Group for Study of Insulin Resistance, and World Health Organization). The authors observed that the frequency of MS in women with RA varied from 16.2% to 40.9% according to the different definitions. However, it was higher than in matched controls (10.5% to 22.9%). Therefore, MS frequency was significantly higher in this series of patients with RA than in healthy controls. When individual components of MS were assessed, hypertension, (*P* < 0.001), high-density lipoprotein cholesterol levels (*P* < 0.001), and abdominal obesity (*P* = 0.019) were found more commonly observed in RA patients than in matched controls. After adjusting for age and physical activity, higher erythrocyte sedimentation rate (OR = 1.52 (95% CI 1.07 to 3.20), *P* = 0.04), disease activity score (OR = 1.74 (95% CI 1.29–2.79), *P* = 0.01), health assessment questionnaire score (OR = 1.58 (95% CI 1.20–2.37), *P* = 0.03), and less methotrexate use (OR = 0.74 (95% CI 0.55–0.96), *P* = 0.02) remained significant independent predictors of the presence of MS in women with RA. Another interesting study by Crowson et al. [19] in 232 patients with RA with no overt CV disease and 1241 non-RA subjects without CV disease showed that RA patients were significantly more likely to have increased waist circumference and elevated blood pressure than non-RA subjects without CV disease. The authors concluded that RA patients were more commonly classified as having MS, and that MS was associated with Health Assessment Questionnaire Disability Index, large-joint swelling, and uric acid levels, but not with C-reactive protein or RA therapies.

Regarding therapy used in the management of RA, Toms et al. [20] showed that methotrexate therapy, unlike other disease modifying antirheumatic drugs (DMARDs) or glucocorticoids, was independently associated with a reduced risk to suffer MS, suggesting a drug-specific mechanism, and making methotrexate a good first-line DMARD in RA patients at high risk of developing MS. In another studies the same authors reported the presence of MS in 40.1% of 398 patients with RA. However, its prevalence did not differ significantly between the different glucocorticoid-exposure groups [29].

Mok et al. [21] assessed the prevalence of the MS in patients with RA, ankylosing spondylitis (AS), and psoriatic arthritis. For this purpose, 930 patients were studied (699 with RA, 122 with AS, and 109 with psoriatic arthritis; 70% women, mean ± standard deviation age 51.1 ± 12.7 years). In this study, the prevalence of MS was significantly higher in psoriatic arthritis (38%) than RA (20%) or AS (11%; *P* < 0.001). The ORs for the MS compared to age- and sex-matched controls were 0.98 (95% CI 0.78–1.23), *P* = 0.88; 0.59 (95% CI 0.30–1.15), *P* = 0.12; and 2.68 (95% CI 1.60–4.50), *P* < 0.001, respectively, for RA, AS, and psoriatic arthritis. Patients with psoriatic arthritis had a significantly higher prevalence of impaired fasting glucose (30%; *P* < 0.001), low HDL cholesterol (33%; *P* < 0.001), high triglycerides level (21%; *P* = 0.008), central obesity (65%; *P* < 0.001), and high blood pressure (56%; *P* = 0.045). They concluded that patients with psoriatic arthritis, but not RA or AS patients, have a significantly higher prevalence of MS compared to the general population.

In another study by Zonana-Nacach et al. [22], 107 RA patients and 85 systemic lupus erythematosus where compared regarding APTIII definitions for MS. They reported that the frequency of obesity and abnormal waist circumference was similar in patients with RA and systemic lupus erythematosus. It was also the case for the frequency of MS in both diseases was also similar (17%). In their series, MS was significantly associated with older age, lower education levels, lower income, and smoking. In RA patients, MS was significantly associated with a shorter period of methotrexate therapy, with pain, and with health assessment questionnaire scores. 

With regard to the relationship between MS and subclinical atherosclerosis in patients with RA, using carotid ultrasonography, Dessein et al. [30] investigated the associations of MS features and MS definitions with common carotid artery intima-media thickness and carotid plaques in 74 RA patients. They concluded that MS was associated with carotid artery intima-media thickness (*P* = 0.04) but not with the presence of carotid plaques (*P* > 0.1). 

There are three exceptions in the literature with regard to the association between MS and RA. With respect to this, using NCEP and WHO criteria, in a case-control study that encompassed 92 RA patients and 96 healthy controls, Karimi et al. [23] did not find differences between groups regarding MS. The only differences observed were a higher frequency of hypertension patients with RA than in the controls and significantly longer duration of the disease in RA patients with MS compared to those without MS. Also, in a series of 200 outpatients with RA and 400 age and sex-matched controls, Karvounaris et al. [25], found a high, albeit comparable to the control population, prevalence of MS in middle-to-older aged patients with RA. However, in this study, in a multivariate logistic regression analysis adjusting for demographics and RA treatment modalities, the risk of having moderate-to-high disease activity (DAS28 > 3.2) was significantly higher in patients with MS than in those without MS components (OR 9.24 (95% CI 1.49–57.2), *P* = 0.02).

Finally, in a series of 120 patients with RA and 431 age- and sex-matched controls study, Sahebari et al. disclosed that the prevalence of IDF or ATPIII MS was significantly higher in controls [24]. In this series the presence of RA was not associated with an increased risk of MS.

A recent study on a random sample of 499 patients with RA disclosed that Vitamin D deficiency was associated independently with an increased risk of hyperlipidemia (OR 1.72 (95% CI 1.10–2.45)) and MS (OR 3.45 (95% CI 1.75–6.80)) in adjusted models [31]. In this regard, another recent study suggested that 25-hydroxyvitamin D may play a protective role against MS in patients with RA [32].

Taking together all these considerations, we can conclude that MS is not uncommon in patients with RA.

## 3. Metabolic Syndrome and Obesity

The underlying cause of the MS is still a challenging question. However, insulin resistance and central obesity are considered to play a key role in the development of MS. Central (abdominal) obesity is independently associated with each of the other MS components and is generally a prerequisite risk factor for the diagnosis of this condition. Although genetics, physical inactivity, ageing, or hormonal changes may also have a causal effect, the chronic and subacute inflammatory state that accompanies obesity has recently been suggested as a potentially unifying pathogenic link [33]. More than a passive storage depot, adipose tissue seems to be a dynamic and metabolically active organ with the ability to elaborate mediators with widespread effects on metabolism, immune function, and vascular homeostasis.

Areas of active investigation focus on the molecular bases of metabolic inflammation and potential pathogenic roles in insulin resistance, diabetes, and CV disease. An increased accumulation of macrophages occurring in obese adipose tissue has emerged as a key process in metabolic inflammation. Recent studies have also begun to unravel the heterogeneity of adipose tissue macrophages, and their physical and functional interactions with adipocytes, endothelial cells, and other immune cells within the adipose tissue microenvironment [33]. 

In conclusion, as shown for insulin resistance, obesity has also a relevant role in the development of MS.

## 4. Obesity and Rheumatoid Arthritis

Obesity is highly prevalent in patients with RA [34]. Changes in body composition have been found in patients with RA, with reduced fat-free mass and increased fat mass and, thus, with little or no weight loss or with a maintained body mass index. This condition has been named “rheumatoid cachexia” and is believed to accelerate morbidity and mortality in RA and has also been linked to MS [35, 36]. 

Abdominal adiposity has been shown to be increased in RA. In a comparative study that included 131 patients with RA and 121 controls, Giles et al. [37] disclosed similar body mass index and waist circumference between both groups. However, the adjusted abdominal visceral fat area was 45 cm^2^ higher (representing a 51% difference) in men with RA patients than in men from the control group (*P* = 0.005) but not significantly different according to RA status in women. In the same study, the adjusted mean abdominal subcutaneous fat area was 119 cm^2^ higher in women with RA (representing a 68% difference) than in women from the control group (*P* < 0.001) but not significantly different according to RA status in men. These authors found that the presence of increased visceral fat area was associated with a significantly higher adjusted probability of having an elevated fasting glucose, hypertension, or the composite definition of the MS for the RA group compared with controls. Within the RA group, rheumatoid factor seropositivity and higher cumulative prednisone exposure were significantly associated with a higher mean adjusted visceral fat area. Higher C-reactive protein levels and lower sharp radiographic scores were significantly associated with both visceral and subcutaneous fat areas. Similar results were described in a report on 80 outpatients with RA [35]. In that study, patients with fat mass index above the 50th percentile and patients with rheumatoid cachexia had the highest frequencies of hypertension and MS. Treatment with glucocorticoids and mean dose given did not differ between those who were cachectic and those not [35]. 

Thus, these studies confirmed the presence of an abnormal body composition in RA and that these abnormalities are related to factors associated with increased CV risk. Most people who are overweight are also *overfat*, but the two are not the same. Overfat is often found in patients with RA. However, Giles et al. [37] showed that the development of MS in patients with RA is due to the presence of a specific altered pattern of fat content. In this regard, for a similar body mass index in patients and controls, the presence of increased visceral fat was associated with a significantly higher adjusted probability of fulfilling the composite definition of the MS in the individuals with RA than in controls. This means the development of MS is not only due to the presence of fat content but the result of a specific pattern of fat deposition.

Obesity but not specifically MS was classically considered to be an important risk factor for the developing RA [38]. However, further studies indicated that obesity is not a strong predisposing factor for RA. Methodological differences and a strict standardization for possible confounders may explain that the association of obesity with RA development had not been confirmed in other studies [39–41].

However, obesity has emerged as a protective risk factor for radiographic joint damage [42]. 

In summary, adiposity and obesity are often present in patients with RA and are associated with increased risk of MS in patients with RA.

## 5. Insulin Resistance and Rheumatoid Arthritis

Insulin resistance is an essential feature of MS that has been linked to RA [43]. In a study that included 94 patients with RA, Dessein and Joffe [44] observed that insulin resistance was associated with markers for inflammation such as C-reactive protein and erythrocyte sedimentation rate and disease activity scores. On the other hand, beta-cell function showed an inverse correlation with DAS28 and swollen and painful joint. Chung et al. [45] studied insulin resistance in 104 patients with RA and compared the results with those of 124 cases of systemic lupus erythematosus. They found that patients with RA have a higher insulin resistance index than systemic lupus erythematosus patients, and that insulin resistance was directly correlated with levels of interleukin 6, tumor necrosis factor-(TNF-) *α*, C-reactive protein, and erythrocyte sedimentation rate or coronary calcification [41]. Others studies have also confirmed the association between insulin resistance and RA [46–49].

In conclusion, insulin resistance is frequently observed in patients with RA.

## 6. Adipokines, Inflammation, and Cardiovascular Risk in Rheumatoid Arthritis

The adipose tissue is a multifunctional organ. Besides the central role of lipid storage, it has a major endocrine function secreting several hormones [50]. These various protein signals have been given the collective name “adipocytokines” or “adipokines.” These molecules are mediators of immune response and inflammation [51]. Adipokines exert potent modulatory actions on target tissues and cells involved in rheumatic disease, including cartilage, synovium, bone, and various immune cells [52]. White adipose tissue-derived cytokines mediate between obesity-related exogenous factors (nutrition and lifestyle) and the molecular events that lead to the development of MS, inflammation, and CV disease [53]. In this regard, a complex adipokine-mediated interaction among white adipose tissue, CV disease, and RA has been described [54]. 

In RA adipocytes and their surrounding macrophages produce a range of adipokines that regulate systemic inflammation [55]. In this regard, the adipokine resistin was initially considered to be only implicated in insulin resistance and type II diabetes mellitus. However, more recent studies have shown that resistin plays an important function in inflammation. Although resistin can be detected at very low levels in human adipose tissue, it is found in peripheral blood mononuclear cells (PBMCs) [56], and resistin gene expression in PBMC is upregulated by proinflammatory cytokines such as TNF-*α* [57]. In some studies, high levels of resistin were found in synovial fluid from patients with RA [58]. However, in other studies plasma resistin levels in RA patients were similar to those found in healthy controls. As pointed out by Gomez et al., this discrepancy may be due to the increased permeability of inflamed synovial membrane in patients with RA [59].

In assessing a series of patients with RA in treatment with the anti-TNF-*α* monoclonal antibody infliximab for severe disease refractory to conventional DMARD therapy including methotrexate, we found positive correlations between markers of inflammation, in particular with C-reactive protein, and resistin levels [60]. Also, TNF-*α* blockade yielded a rapid reduction in the levels of resistin in these patients [60]. These observations support a potential role of resistin in the inflammatory cascade in RA.

Adiponectin, another important adipokine, is an especially promising candidate in explaining the link between obesity, metabolism, and systemic inflammation [54, 61]. Low circulating adiponectin concentrations constitute an MS feature and circulating adiponectin has antiinflammatory, antiatherogenic, and antidiabetic properties [62, 63]. In patients with RA undergoing anti-TNF-*α* therapy due to severe disease high-grade inflammation was independently and negatively correlated with circulating adiponectin concentrations [64]. Low adiponectin concentrations further clustered and correlated with MS features such as dyslipidemia and high plasma glucose that have been reportedly to contribute to the accelerated atherogenesis of patients with RA [64]. These findings may suggest that low circulating adiponectin levels may be implicated in the development of CV disease associated to RA. However, the interaction of high-grade inflammation with low circulating adiponectin concentrations does not likely to be TNF-*α* mediated in RA [64]. In this regard, no association between adiponectin and carotid intima-media wall thickness, a surrogate marker of CV events in RA [65], was observed in patients with RA [66]. In keeping with these negative results, no association between functional adiponectin—ADIPOQ rs266729 and ADIPOQ rs1501299 polymorphisms—and CV disease was found in patients with RA [67].

Leptin is another important adipokine. This peptide plays an important role in the regulation of body weight by inhibiting food intake and stimulating energy expenditure. Leptin is also a proinflammatory adipocyte-derived factor that operates in the cytokine network by linking immune and inflammatory processes to the neuroendocrine system [68, 69]. Leptin acts as a modulator of T-cell activity and plays a key role in some autoimmune inflammatory diseases such as type 1 diabetes [70]. This adipokine is produced by stimulation of inflammatory cytokines such as TNF-*α* and interleukin-1. Importantly, leptin exerts many potential atherogenic effects and high leptin concentrations predict incident CV disease in non-RA subjects [71]. Recent studies have shown that high leptin levels may play an important role in the development of CV disease associated to obesity including atherosclerosis. Leptin exerts many atherogenic effects such as induction of endothelial dysfunction, stimulation of inflammatory reaction, oxidative stress, reduction of paraoxonase activity, platelet aggregation, migration, hypertrophy, and proliferation of vascular smooth muscle cells [71]. 

In patients with RA, circulating leptin levels have been described as either higher or unmodified in comparison to healthy controls [54, 69]. In patients with RA undergoing anti-TNF-*α* therapy, a correlation between serum leptin levels and VCAM-1 was observed [72]. This is of potential interest as biomarkers of endothelial dysfunction-endothelial cell activation have been found elevated in patients with RA and anti-TNF-*α* therapy improved endothelial dysfunction [73, 74] and led to a reduction of the levels of some of these endothelial cell activation biomarkers [75]. However, no immediate change in serum leptin levels upon anti-TNF-*α*-infliximab infusion was observed [72]. These results were in keeping with those obtained by other investigators who found an absence of change plasma leptin concentrations after several weeks' treatment with TNF-*α* blockers [76, 77]. 

It is known that MS features are independently associated with atherosclerosis in RA [17, 30]. However, in contrast to what was reported in non-RA subjects, only body mass index but not insulin resistance, blood pressure, or the lipid profile was related to leptin concentrations in patients with severe RA [72].

A potential association between the adipokine visfatin, also called pre-B-cell colony-enhancing factor, and inflammation has been proposed in the last years. Circulating levels of visfatin are correlated with the amount of visceral fat [78], even though visfatin is produced also by endotoxin-stimulated neutrophils. Also, visfatin synthesis is regulated by numerous factors, among other corticosteroids, TNF-*α*, interleukin-6, and growth hormone [79]. Otero et al. showed higher circulating visfatin levels in patients with RA in comparison to healthy subjects [54]. However, up to now it cannot be excluded the direct effect of different proinflammatory factors in the production of visfatin in patients with RA. Haider et al. [80] demonstrated in young healthy nonobese subjects, that visfatin concentrations are increased by hyperglycemia, and this effect was prevented by coinfusion of insulin or somatostatin. These findings may have high relevance in patients with RA who often present a MS that included insulin resistance, which, in turn, is improved following TNF-*α* blockade [81]. However, in a study that included RA patients with severe disease undergoing anti-TNF-*α*-infliximab therapy visfatin levels were not associated with inflammation or MS and infliximab infusion did not yield significant changes in visfatin levels [82].

In summary, an abnormal adipokine profile is often found in patient with RA.

## 7. Effect of Anti-TNF-***α*** Therapy on Insulin Resistance in Patients with Rheumatoid Arthritis 

A disturbed glucose metabolism is included within the constellation of CV risk factors of the MS. In patients with RA insulin resistance is closely related to the presence of a chronic proinflammatory state [41]. In the general population TNF-*α* production is increased under chronic hyperglycemia and TNF-*α* has negative effect on insulin sensitivity [83]. TNF-*α* is also an important mediator of insulin resistance in obesity and diabetes through its ability to decrease the tyrosine kinase activity of the insulin receptor. This pivotal proinflammatory cytokine also impedes insulin-glucose-mediated uptake in the skeletal muscle [84]. 

In a cross-sectional study using acute phase responses as surrogate markers of systemic inflammation, Dessein et al. disclosed a consistent association with insulin resistance in patients with RA [85]. In their series of patients with RA the impaired insulin sensitivity was significantly associated with both low HDL cholesterol and high triglycerides, which are abnormalities that reflect the lipid component of atherogenic dyslipidemia observed in this chronic inflammatory disease [85]. In a further longitudinal study, the same investigators found that the initiation of DMARDs in combination with pulsed corticosteroids, which were employed only at the onset and as bridge therapy to accelerate the DMARD response, yielded an improvement of insulin sensitivity and a reduction in atherogenic dyslipidemia [86]. Therefore, these results reinforce the claim that high-grade systemic inflammation in RA clearly clustered with insulin resistance and its suppression was associated with an improvement of the MS in which insulin resistance has an important role [86]. 

In line with the above, several studies have also assessed the effect of the TNF-*α* antagonists on the mechanisms associated with accelerated atherogenesis in RA, including specifically the effect of these biologic agents on insulin resistance. These studies have been conducted using the most common commercially available TNF-*α* antagonists that neutralize TNF-*α* in patients with severe RA refractory to conventional DMARD therapy. The first studies were performed using infliximab, a chimeric IgG1 *α* monoclonal antibody binding TNF-*α*. Some others were performed using etanercept, a protein composed of two p75 TNF-*α* receptors fused to the Fc portion of IgG1. Less frequently, the TNF-*α* antagonist used to undertake the study was adalimumab, a fully humanized IgG1*α* monoclonal antibody [87].

We observed an immediate reduction in the serum insulin levels following infliximab infusion in most patients undergoing this biologic therapy because severe RA refractory to DMARD therapy [81]. Moreover, statistically significant reduction in the insulin/glucose index was observed. Most patients experienced improvement of insulin resistance manifested by a decrease in the Homeostasis Model Assessment of Insulin Resistance (HOMA) index. Moreover, a significant improvement of insulin sensitivity was also observed in most patients [81].

Several studies were also conducted to determine long-term effect of TNF-*α* antagonists on insulin resistance [87]. These studies assessed the effect of these biologic agents on insulin resistance in patients undergoing TNF-*α* blocker therapy after several weeks or months of periodical treatment with these drugs. In most cases, insulin resistance was assessed by the HOMA and the Quantitative Insulin Sensitivity Check Index (QUICKI), as previously described [81]. In one of them, Kiortsis et al. showed reduction in HOMA and increase of QUICKI in the subgroup of RA patients with initially had the highest tertile of insulin resistance [88]. In a prospective study of RA patients with active disease, Seriolo et al. observed a significant decrease of the HOMA index and increase of QUICKI in infliximab-treated RA patients for 24 weeks [89]. Similar improvement of HOMA has been shown by other authors [90]. Also, additional evidence using hyperinsulinemic euglycemic clamps in nondiabetic RA patients was observed [91]. A recent study has confirmed that anti-TNF-*α* therapy improves insulin resistance, beta-cell function, and reverted defects in the insulin signaling cascade in active RA patients with high insulin resistance [92]. In this study that included patients treated with different TNF-*α* blockers, mainly with infliximab, an improvement of insulin sensitivity was also observed [92].

Interestingly, as previously described [81], the improvement of insulin sensitivity correlated negatively with the baseline BMI [91]. With respect to this, a recent report has shown that the improvement of insulin resistance in patients with RA undergoing anti-TNF-*α* therapy is impaired by the presence of obesity [93]. In this regard, obesity at the onset of treatment with TNF-*α* blockers leads to a reduction of the beneficial effect of these drugs on MS associated to RA. Therefore, weight-loss and exercise should be considered in the management of RA patients undergoing anti-TNF-*α* blockers to improve the effect of these drugs on insulin sensitivity [94].

Taking together all these considerations, anti-TNF-*α* therapy exerts beneficial metabolic effects by the reduction of insulin resistance and improvement of insulin sensitivity.

## 8. Conclusion

In light of our paper, there seems to be ample evidence supporting a relationship between MS and RA. Understanding how inflammation arising in one tissue affects the physiology and pathology of other organs remains an unanswered question with therapeutic implications for chronic conditions including obesity, diabetes mellitus, atherosclerosis, and RA.

Adipokines may influence the development of atherogenesis in these patients. Biologic therapies that block proinflammatory cytokines seem to have beneficial effects on the insulin resistance observed in patients with RA.

## Figures and Tables

**Table 1:** Five current definitions of metabolic syndrome.

Required	NCEP ATP3 2005	IDF 2006 Waist = 94 cm (men) or = 80 cm (women)	EGIR 1999 Insulin resistance or fasting hyperinsulinemia in top 25 percent	WHO 1999	AACE 2003 High risk of insulin resistance; or BMI = 25 kg/m^2^; or waist = 102 cm (men) or = 88 cm (women)
				Insulin resistance in top 25 percent;	
				glucose = 6.1 mmol/L (110 mg/dL);	
				2-hour glucose = 7.8 mmol/L (140 mg/dL)	
Number of abnormalities	3	2	2	2	2
Glucose	mmol/L (100 mg/dL)	5.6 mmol/L (100 mg/dL)	6.1–6.9 mmol/L (110–125 mg/dL)		6.1 mmol/L (110 mg/dL);
	or drug treatment for elevated blood glucose	or diagnosed diabetes			2-hour glucose 7.8 mmol/L (140 mg/dL)
HDL cholesterol	<1.0 mmol/L (40 mg/dL) (men);	<1.0 mmol/L (40 mg/dL) (men);	<1.0 mmol/L (40 mg/dL)	<0.9 mmol/L (35 mg/dL) (men); <1.0 mmol/L (40 mg/dL) (women)	<1.0 mmol/L (40 mg/dL) (men);
	<1.3 mmol/L (50 mg/dL) (women);	<1.3 mmol/L (50 mg/dL) (women);			<1.3 mmol/L (50 mg/dL) (women)
	or drug treatment for low HDL-C	or drug treatment for low HDL-C			
Triglycerides	1.7 mmol/L (150 mg/dL)	1.7 mmol/L (150 mg/dL)	2.0 mmol/L (180 mg/dL)	1.7 mmol/L (150 mg/dL)	1.7 mmol/L (150 mg/dL)
	or drug treatment for elevated triglycerides	or drug treatment for high triglycerides	or drug treatment for dyslipidemia		
Obesity	Waist = 102 cm (men)		Waist = 94 cm (men)	Waist/hip ratio > 0.9 (men)	
	or = 88 cm (women)		or = 80 cm (women)	or >0.85 (women)	
				or BMI = 30 kg/m^2^	
Hypertension	130/85 mmHg	130/85 mmHg	140/90 mmHg	140/90 mmHg	130/85 mmHg
	or drug treatment for hypertension	or drug treatment for hypertension	or drug treatment for hypertension		

NCEP: National Cholesterol Education Program; IDF: International Diabetes Federation; EGIR: Group for the Study of Insulin Resistance; WHO: World Health Organization; AACE: American Association of Clinical Endocrinologists.

HDL-C: high-density lipoprotein cholesterol, LDL: low-density lipoprotein cholesterol, BMI: body mass index.

**Table 2:** Metabolic syndrome and rheumatoid arthritis.

Reference	RA/controls	Association	MS definition used	Comments
Da Cunha et al. [16]	283/226	Yes	NCEP	MS associated with disease activity. Increased prevalence of waist circumference, blood pressure, and fasting glucose in this RA population when compared to controls.
Chung et al. [17]	154/85	Yes	NCEP, WHO	88 with early RA and 66 with long-standing RA.
				RA patients with MS had an increased risk of having higher coronary-artery calcification score;
				this association of RA and MS has been also observed when early RA was considered.
Dao et al. [18]	105/105	Yes	NCEP, WHO	Early RA already had higher prevalence of MS compared with healthy controls.
			IDF, EGIR	Higher systemic inflammatory markers, disease activity and disability scores, and less methotrexate use were independent predictors associated with the presence of MS in women with early RA.
Crowson et al. [19]	232/1241	Yes	NCEP	Only RA patients with no overt cardiovascular disease were considered.
				RA patients were significantly more likely to have increased waist circumference and elevated blood pressure than non-RA subjects
				Significantly more RA patients were classified as having MS.
Toms et al. [20]	400/—	Yes	NCEP, WHO	MS prevalence rates varied from 12.1% to 45.3% in RA according to the definition used.
			IDF, EGIR	Methotrexate use, but not other DMARDs or glucocorticoids, was associated with significantly reduced chance of having MS in RA.
Mok et al. [21]	699 RA	No	Asian criteriafor central obesity	The prevalence of MS was significantly higher in PsA (38%) than RA (20%) or AS (11%; *P* < 0.001).
	122 AS			Patients with PsA, but not those with RA or AS, have a significantly higher prevalence of MS compared to the general population.
	109 PsA			In RA patients, MS was related to pain and functional status, indicating disease activity.
Zonana-Nacach et al. [22]	107 RA/85 LES	Yes	NCEP	The frequency of MS in RA and SLE patients was similar and associated with smoking.
Karimi et al. [23]	92/96	No	NCEP, WHO	The duration of RA was associated with MS.
Sahebari et al. [24]	120/431	No	NCEP, IDF	The prevalence of MS was significantly higher in the control group.
				There was no association between the DAS28 and the presence of MS components by either definition.
Karvounaris et al. [25]	200/400	No	NCEP	Risk of having moderate-to-high disease activity (DAS28 > 3.2) was significantly higher in patients with MS compared with those with no MS components.

NCEP: National Cholesterol Education Program; IDF: International Diabetes Federation; EGIR: Group for the Study of Insulin Resistance; WHO: World Health Organization.

MS: metabolic syndrome, DAS28: disease activity score, RA: rheumatoid arthritis, SLE: systemic lupus erythematosus, DMARDs: disease modifying antirheumatic drugs.

PsA: psoriatic arthritis, AS: ankylosing spondylitis.

The term Association means if an association between RA and MS was observed.

Toms, Mok and Zonana did not study healthy controls.

## References

[1] Kahn R, Buse J, Ferrannini E, Stern M (2005). The metabolic syndrome: time for a critical appraisal—joint statement from the American Diabetes Association and the European Association for the Study of Diabetes. *Diabetes Care*.

[2] Bayturan O, Tuzcu EM, Lavoie A (2010). The metabolic syndrome, its component risk factors, and progression of coronary atherosclerosis. *Archives of Internal Medicine*.

[3] Isomaa B, Almgren P, Tuomi T (2001). Cardiovascular morbidity and mortality associated with the metabolic syndrome. *Diabetes Care*.

[4] Dunstan DW, Zimmet PZ, Welborn TA (2002). The rising prevalence of diabetes and impaired glucose tolerance: the Australian diabetes, obesity and lifestyle study. *Diabetes Care*.

[5] Stern MP, Williams K, González-Villalpando C, Hunt KJ, Haffner SM (2004). Does the metabolic-syndrome improve identification of individuals at risk of type 2 diabetes and/or cardiovascular disease?. *Diabetes Care*.

[6] Hamaguchi M, Kojima T, Takeda N (2005). The metabolic syndrome as a predictor of nonalcoholic fatty liver disease. *Annals of Internal Medicine*.

[7] Chen J, Muntner P, Hamm LL (2004). The metabolic syndrome and chronic kidney disease in U.S. adults. *Annals of Internal Medicine*.

[8] Pasquali R, Gambineri A, Anconetani B (1999). The natural history of the metabolic syndrome in young women with the polycystic ovary syndrome and the effect of long-term oestrogen-progestagen treatment. *Clinical Endocrinology*.

[9] Ip MSM, Lam B, Ng MMT, Lam WK, Tsang KWT, Lam KSL (2002). Obstructive sleep apnea is independently associated with insulin resistance. *American Journal of Respiratory and Critical Care Medicine*.

[10] Choi HK, Ford ES, Li C, Curhan G (2007). Prevalence of the metabolic syndrome in patients with gout: the Third National Health and Nutrition Examination Survey. *Arthritis Care and Research*.

[11] Alberti KGMM, Zimmet P, Shaw J (2005). The metabolic syndrome—a new worldwide definition. *The Lancet*.

[12] Grundy SM, Cleeman JI, Daniels SR (2005). Diagnosis and management of the metabolic syndrome: an American Heart Association/National Heart, Lung, and Blood Institute scientific statement. *Circulation*.

[13] Genuth S, Alberti KG, Bennett P (2003). Follow-up report on the diagnosis of diabetes mellitus. *Diabetes Care*.

[14] Cleeman JI (2001). Executive summary of the third report of the National Cholesterol Education Program (NCEP) expert panel on detection, evaluation, and treatment of high blood cholesterol in adults (adult treatment panel III). *Journal of the American Medical Association*.

[15] Meigs JB, Rutter MK, Sullivan LM, Fox CS, D’Agostino RB, Wilson PWF (2007). Impact of insulin resistance on risk of type 2 diabetes and cardiovascular disease in people with metabolic syndrome. *Diabetes Care*.

[16] Da Cunha VR, Brenol CV, Brenol JCT (2012). Metabolic syndrome prevalence is increased in rheumatoid arthritis patients and is associated with disease activity. *Scandinavian Journal of Rheumatology*.

[17] Chung CP, Oeser A, Solus JF (2008). Prevalence of the metabolic syndrome is increased in rheumatoid arthritis and is associated with coronary atherosclerosis. *Atherosclerosis*.

[18] Dao HH, Do QT, Sakamoto J (2010). Increased frequency of metabolic syndrome among Vietnamese women with early rheumatoid arthritis: a cross-sectional study. *Arthritis Research and Therapy*.

[19] Crowson CS, Myasoedova E, Davis JM (2011). Increased prevalence of metabolic syndrome associated with rheumatoid arthritis in patients without clinical cardiovascular disease. *Journal of Rheumatology*.

[20] Toms TE, Panoulas VF, John H, Douglas KM, Kitas GD (2009). Methotrexate therapy associates with reduced prevalence of the metabolic syndrome in rheumatoid arthritis patients over the age of 60- more than just an anti-inflammatory effect? A cross sectional study. *Arthritis Research & Therapy*.

[21] Mok CC, Ko GTC, Ho LY, Yu KL, Chan PT, To CH (2011). Prevalence of atherosclerotic risk factors and the metabolic syndrome in patients with chronic inflammatory arthritis. *Arthritis Care and Research*.

[22] Zonana-Nacach A, Santana-Sahagún E, Jiménez-Balderas FJ, Camargo-Coronel A (2008). Prevalence and factors associated with metabolic syndrome in patients with rheumatoid arthritis and systemic lupus erythematosus. *Journal of Clinical Rheumatology*.

[23] Karimi M, Mazloomzadeh S, Kafan S, Amirmoghadami H (2011). The frequency of metabolic syndrome in women with rheumatoid arthritis and in controls. *International Journal of Rheumatic Diseases*.

[24] Sahebari M, Goshayeshi L, Mirfeizi Z (2011). Investigation of the association between metabolic syndrome and disease activity in rheumatoid arthritis. *TheScientificWorldJournal*.

[25] Karvounaris SA, Sidiropoulos PI, Papadakis JA (2007). Metabolic syndrome is common among middle-to-older aged Mediterranean patients with rheumatoid arthritis and correlates with disease activity: a retrospective, cross-sectional, controlled, study. *Annals of the Rheumatic Diseases*.

[26] Meune C, Touzé E, Trinquart L, Allanore Y (2009). Trends in cardiovascular mortality in patients with rheumatoid arthritis over 50 years: a systematic review and meta-analysis of cohort studies. *Rheumatology*.

[27] Aviña-Zubieta JA, Choi HK, Sadatsafavi M, Etminan M, Esdaile JM, Lacaille D (2008). Risk of cardiovascular mortality in patients with rheumatoid arthritis: a meta-analysis of observational studies. *Arthritis Care and Research*.

[28] Cavagna L, Boffini N, Cagnotto G, Inverardi F, Grosso V, Caporali R (2012). Atherosclerosis and rheumatoid arthritis: more than a simple association. *Mediators of Inflammation*.

[29] Toms TE, Panoulas VF, Douglas KMJ, Griffiths HR, Kitas GD (2008). Lack of association between glucocorticoid use and presence of the metabolic syndrome in patients with rheumatoid arthritis: a cross-sectional study. *Arthritis Research and Therapy*.

[30] Dessein PH, Tobias M, Veller MG (2006). Metabolic syndrome and subclinical atherosclerosis in rheumatoid arthritis. *Journal of Rheumatology*.

[31] Baker JF, Mehta NN, Baker DG (2012). Vitamin D, metabolic dyslipidemia, and metabolic syndrome in rheumatoid arthritis. *American Journal of Medicine*.

[32] Goshayeshi L, Saber H, Sahebari M (2012). Association between metabolic syndrome, BMI, and serum vitamin D concentrations in rheumatoid arthritis. *Clinical Rheumatology*.

[33] Romeo GR, Lee J, Shoelson SE (2012). Metabolic syndrome, insulin resistance, and roles of inflammation- mechanisms and therapeutic targets. *Arteriosclerosis, Thrombosis, and Vascular Biology*.

[34] Stavropoulos-Kalinoglou A, Metsios GS, Koutedakis Y, Kitas GD (2011). Obesity in rheumatoid arthritis. *Rheumatology*.

[35] Elkan AC, Håkansson N, Frostegård J, Cederholm T, Hafström I (2009). Rheumatoid cachexia is associated with dyslipidemia and low levels of atheroprotective natural antibodies against phosphorylcholine but not with dietary fat in patients with rheumatoid arthritis: a cross-sectional study. *Arthritis Research and Therapy*.

[36] Roubenoff R (2009). Rheumatoid cachexia: a complication of rheumatoid arthritis moves into the 21st century. *Arthritis Research and Therapy*.

[37] Giles JT, Allison M, Blumenthal RS (2010). Abdominal adiposity in rheumatoid arthritis: association with cardiometabolic risk factors and disease characteristics. *Arthritis and Rheumatism*.

[38] Derdemezis CS, Voulgari PV, Drosos AA (2011). Obesity, adipose tissue and rheumatoid arthritis: coincidence or more complex relationship?. *Clinical and Experimental Rheumatology*.

[39] Cerhan JR, Saag KG, Criswell LA, Merlino LA, Mikuls TR (2002). Blood transfusion, alcohol use, and anthropometric risk factors for rheumatoid arthritis in older women. *Journal of Rheumatology*.

[40] Bartfai T, Waalen J, Buxbaum JN (2007). Adipose tissue as a modulator of clinical inflammation: does obesity reduce the prevalence of rheumatoid arthritis?. *Journal of Rheumatology*.

[41] Rodriguez LA, Tolosa LB, Ruigmez A, Johansson S, Wallander MA (2009). Rheumatoid arthritis in UK primary care incidence and prior morbidity. *Scandinavian Journal of Rheumatology*.

[42] Van Der Helm-van Mil AHM, Van Der Kooij SM, Allaart CF, Toes REM, Huizinga TWJ (2008). A high body mass index has a protective effect on the amount of joint destruction in small joints in early rheumatoid arthritis. *Annals of the Rheumatic Diseases*.

[43] Ferraz Amaro I, Díaz González F, González Juanatey C, González Gay MA (2011). Insulin resistance and rheumatoid arthritis. *Reumatologia Clinica*.

[44] Dessein PH, Joffe BI (2006). Insulin resistance and impaired beta cell function in rheumatoid arthritis. *Arthritis and Rheumatism*.

[45] Chung CP, Oeser A, Solus JF (2008). Inflammation-associated insulin resistance: differential effects in rheumatoid arthritis and systemic lupus erythematosus define potential mechanisms. *Arthritis and Rheumatism*.

[46] La Montagna G, Cacciapuoti F, Buono R (2007). Insulin resistance is an independent risk factor for atherosclerosis in rheumatoid arthritis. *Diabetes and Vascular Disease Research*.

[47] Dessein PH, Norton GR, Woodiwiss AJ, Joffe BI, Solomon A (2007). Independent role of conventional cardiovascular risk factors as predictors of c-reactive protein concentrations in rheumatoid arthritis. *Journal of Rheumatology*.

[48] Pamuk ON, Unlu E, Cakir N (2006). Role of insulin resistance in increased frequency of atherosclerosis detected by carotid ultrasonography in rheumatoid arthritis. *Journal of Rheumatology*.

[49] Dessein PH, Joffe BI, Stanwix A, Botha AS, Moomal Z (2002). The acute phase response does not fully predict the presence of insulin resistance and dyslipidemia in inflammatory arthritis. *Journal of Rheumatology*.

[50] Trayhurn P, Wood IS (2004). Adipokines: inflammation and the pleiotropic role of white adipose tissue. *British Journal of Nutrition*.

[51] Lago F, Dieguez C, Gómez-Reino J, Gualillo O (2007). Adipokines as emerging mediators of immune response and inflammation. *Nature Clinical Practice Rheumatology*.

[52] Gómez R, Conde J, Scotece M, Gómez-Reino JJ, Lago F, Gualillo O (2011). What's new in our understanding of the role of adipokines in rheumatic diseases?. *Nature Reviews Rheumatology*.

[53] Gualillo O, González-Juanatey JR, Lago F (2007). The emerging role of adipokines as mediators of cardiovascular function: physiologic and clinical perspectives. *Trends in Cardiovascular Medicine*.

[54] Otero M, Logo R, Gomez R (2006). Changes in plasma levels of fat-derived hormones adiponectin, leptin, resistin and visfatin in patients with rheumatoid arthritis. *Annals of the Rheumatic Diseases*.

[55] Scotece M, Conde J, Gómez R (2012). Role of adipokines in atherosclerosis: interferences with cardiovascular complications in rheumatic diseases. *Mediators of Inflammation*.

[56] Patel L, Buckels AC, Kinghorn IJ (2003). Resistin is expressed in human macrophages and directly regulated by PPAR*γ* activators. *Biochemical and Biophysical Research Communications*.

[57] Kaser S, Kaser A, Sandhofer A, Ebenbichler CF, Tilg H, Patsch JR (2003). Resistin messenger-RNA expression is increased by proinflammatory cytokines in vitro. *Biochemical and Biophysical Research Communications*.

[58] Schäffler A, Ehling A, Neumann E (2003). Adipocytokines in synovial fluid. *Journal of the American Medical Association*.

[59] Gomez R, Lago F, Gomez-Reino J, Dieguez C, Gualillo O (2009). Adipokines in the skeleton: influence on cartilage function and joint degenerative diseases. *Journal of Molecular Endocrinology*.

[60] Gonzalez-Gay MA, Garcia-Unzueta MT, Gonzalez-Juanatey C (2008). Anti-TNF-*α* therapy modulates resistin in patients with rheumatoid arthritis. *Clinical and Experimental Rheumatology*.

[61] Conde J, Scotece M, Gómez R (2011). Adipokines: biofactors from white adipose tissue. A complex hub among inflammation, metabolism, and immunity. *BioFactors*.

[62] Ouchi N, Kihara S, Arita Y (1999). Novel modulator for endothelial adhesion molecules: adipocyte-derived plasma protein adiponectin. *Circulation*.

[63] Ryo M, Nakamura T, Kihara S (2004). Adiponectin as a biomarker of the metabolic syndrome. *Circulation Journal*.

[64] Gonzalez-Gay MA, Llorca J, Garcia-Unzueta MT (2008). High-grade inflammation, circulating adiponectin concentrations and cardiovascular risk factors in severe rheumatoid arthritis. *Clinical and Experimental Rheumatology*.

[65] Gonzalez-Juanatey C, Llorca J, Martin J, Gonzalez-Gay MA (2009). Carotid intima-media thickness predicts the development of cardiovascular events in patients with rheumatoid arthritis. *Seminars in Arthritis and Rheumatism*.

[66] Gonzalez-Gay MA, Gonzalez-Juanatey C, Rodriguez-Rodriguez L, Miranda-Filloy JA, Martin J, Llorca J (2011). Lack of association between adipokines and ghrelin and carotid intima-media thickness in patients with severe rheumatoid arthritis. *Clinical and Experimental Rheumatology*.

[67] Rodríguez-Rodríguez L, García-Bermúdez M, González-Juanatey C (2011). Lack of association between ADIPOQ rs266729 and ADIPOQ rs1501299 polymorphisms and cardiovascular disease in rheumatoid arthritis patients. *Tissue Antigens*.

[68] Tilg H, Moschen AR (2006). Adipocytokines: mediators linking adipose tissue, inflammation and immunity. *Nature Reviews Immunology*.

[69] Scotece M, Conde J, Gómez R (2011). Beyond fat mass: exploring the role of adipokines in rheumatic diseases. *TheScientificWorldJournal*.

[70] Otero M, Lago R, Gómez R, Lago F, Gómez-Reino JJ, Gualillo O (2006). Leptin: a metabolic hormone that functions like a proinflammatory adipokine. *Drug News and Perspectives*.

[71] Beltowski J (2006). Leptin and atherosclerosis. *Atherosclerosis*.

[72] Gonzalez-Gay MA, Garcia-Unzueta MT, Berja A (2009). Anti-TNF-*α* therapy does not modulate leptin in patients with severe rheumatoid arthritis. *Clinical and Experimental Rheumatology*.

[73] Gonzalez-Juanatey C, Llorca J, Sanchez Andrade A, Garcia-Porrua C, Martin J, Gonzalez-Gay MA (2006). Short-term adalimumab therapy improves endothelial function in patients with rheumatoid arthritis refractory to infliximab. *Clinical and Experimental Rheumatology*.

[74] Gonzalez-Juanatey C, Vazquez-Rodriguez TR, Gomez-Acebo I (2012). Anti-TNF-alpha-adalimumab therapy is associated with persistent improvement of endothelial function without progression of carotid intima-media wall thickness in patients with rheumatoid arthritis refractory to conventional therapy. *Mediators of Inflammation*.

[75] Gonzalez-Gay MA, Garcia-Unzueta MT, De Matias JM (2006). Influence of anti-TNF-*α* infliximab therapy on adhesion molecules associated with atherogenesis in patients with rheumatoid arthritis. *Clinical and Experimental Rheumatology*.

[76] Popa C, Netea MG, Radstake TRDS, Van Riel PL, Barrera P, Van Der Meer JWM (2005). Markers of inflammation are negatively correlated with serum leptin in rheumatoid arthritis. *Annals of the Rheumatic Diseases*.

[77] Härle P, Sarzi-Puttini P, Cutolo M, Straub RH (2006). No change of serum levels of leptin and adiponectin during anti-tumour necrosis factor antibody treatment with adalimumab in patients with rheumatoid arthritis. *Annals of the Rheumatic Diseases*.

[78] Fukuhara A, Matsuda M, Nishizawa M (2005). Visfatin: a protein secreted by visceral fat that Mimics the effects of insulin. *Science*.

[79] Berndt J, Klöting N, Kralisch S (2005). Plasma visfatin concentrations and fat depot-specific mRNA expression in humans. *Diabetes*.

[80] Haider DG, Schaller G, Kapiotis S, Maier C, Luger A, Wolzt M (2006). The release of the adipocytokine visfatin is regulated by glucose and insulin. *Diabetologia*.

[81] Gonzalez-Gay MA, De Matias JM, Gonzalez-Juanatey C (2006). Anti-tumor necrosis factor-*α* blockade improves insulin resistance in patients with rheumatoid arthritis. *Clinical and Experimental Rheumatology*.

[82] Gonzalez-Gay MA, Vazquez-Rodriguez TR, Garcia-Unzueta MT (2010). Visfatin is not associated with inflammation or metabolic syndrome in patients with severe rheumatoid arthritis undergoing anti-TNF-*α* therapy. *Clinical and Experimental Rheumatology*.

[83] Fukuzawa M, Satoh J, Qiang X (1999). Inhibition of tumor necrosis factor-*α* with anti-diabetic agents. *Diabetes Research and Clinical Practice*.

[84] Hotamisligil GS, Peraldi P, Budavari A, Ellis R, White MF, Spiegelman BM (1996). IRS-1-mediated inhibition of insulin receptor tyrosine kinase activity in TNF-*α*- and obesity-induced insulin resistance. *Science*.

[85] Dessein PH, Stanwix AE, Joffe BI (2002). Cardiovascular risk in rheumatoid arthritis versus osteoarthritis: acute phase response related decreased insulin sensitivity and high-density lipoprotein cholesterol as well as clustering of metabolic syndrome features in rheumatoid arthritis. *Arthritis Research*.

[86] Dessein PH, Joffe BI, Stanwix AE (2002). Effects of disease modifying agents and dietary intervention on insulin resistance and dyslipidemia in inflammatory arthritis: a pilot study. *Arthritis Research*.

[87] Gonzalez-Gay MA, Gonzalez-Juanatey C, Vazquez-Rodriguez TR, Miranda-Filloy JA, Llorca J (2010). Insulin resistance in rheumatoid arthritis: the impact of the anti-TNF-*α* therapy: annals of the New York Academy of Sciences. *Annals of the New York Academy of Sciences*.

[88] Kiortsis DN, Mavridis AK, Vasakos S, Nikas SN, Drosos AA (2005). Effects of infliximab treatment on insulin resistance in patients with rheumatoid arthritis and ankylosing spondylitis. *Annals of the Rheumatic Diseases*.

[89] Seriolo B, Paolino S, Ferrone C, Cutolo M (2008). Impact of long-term anti-TNF-*α* treatment on insulin resistance in patients with rheumatoid arthritis. *Clinical and Experimental Rheumatology*.

[90] Tam LS, Tomlinson B, Chu TT, Li TK, Li EK (2007). Impact of TNF inhibition on insulin resistance and lipids levels in patients with rheumatoid arthritis. *Clinical Rheumatology*.

[91] Huvers FC, Popa C, Netea MG, Van Den Hoogen FHJ, Tack CJ (2007). Improved insulin sensitivity by anti-TNF*α* antibody treatment in patients with rheumatic diseases. *Annals of the Rheumatic Diseases*.

[92] Stagakis  I, Bertsias  G, Karvounaris S (2012). Anti-tumor necrosis factor therapy improves insulin resistance, beta cell function and insulin signaling in active rheumatoid arthritis patients with high insulin resistance. *Arthritis Research and Therapy*.

[93] Stavropoulos-Kalinoglou A, Metsios GS, Panoulas VF (2012). Anti-tumour necrosis factor alpha therapy improves insulin sensitivity in normal-weight but not in obese patients with rheumatoid arthritis. *Arthritis Research & Therapy*.

[94] González-Gay MA, González-Juanatey C (2012). Rheumatoid arthritis: obesity impairs efficacy of anti-TNF therapy in patients with RA. *Nature Reviews Rheumatology*.

